# Urinary Cortisol Increases During a Respiratory Outbreak in Wild Chimpanzees

**DOI:** 10.3389/fvets.2020.00485

**Published:** 2020-08-21

**Authors:** Verena Behringer, Anna Preis, Doris F. Wu, Catherine Crockford, Fabian H. Leendertz, Roman M. Wittig, Tobias Deschner

**Affiliations:** ^1^Max Planck Institute for Evolutionary Anthropology, Leipzig, Germany; ^2^Endocrinology Laboratory, German Primate Center, Leibniz Institute for Primate Research, Göttingen, Germany; ^3^Epidemiology of Highly Pathogenic Microorganisms, Robert Koch Institute, Berlin, Germany; ^4^Taï Chimpanzee Project, Centre Suisse de Recherches Scientifiques en Côte d'Ivoire, Abidjan, Côte d'Ivoire

**Keywords:** disease monitoring, non-invasive, *pan troglodytes*, ecoimmunology, costly immune responses

## Abstract

**Abstract:** In mammals, the excretion of cortisol can provide energy toward restoring homeostasis and is a major component of the stress response. However, chronically elevated cortisol levels also have suppressive effects on immune function. As mounting an immune response is energetically costly, sick individuals may conserve energy by exhibiting certain sickness behaviors, such as declining activity levels. Due to the complex interplay between immune function and sickness behaviors, endocrinological correlates have received growing attention in the medical community, but so far, this subject was investigated rarely. Furthermore, given the complexities of studying illnesses and immunity in natural settings, correlates of sickness behaviors have yet to be studied in non-human primates in the wild.

**Methods:** We measured urinary cortisol levels using liquid chromatography–mass spectrometry in a group of wild habituated chimpanzees in Taï National Park, Côte d'Ivoire, before, during, and after a respiratory disease outbreak (main causative pathogen: human respiratory syncytial virus A, with coinfections of *Streptococcus pneumoniae*). Changes in cortisol levels were then related to urinary neopterin levels, a biomarker of immune system activation.

**Results:** Urinary cortisol levels were found to be more than 10-fold higher during the outbreak in comparison with levels before and after the outbreak period. Increasing cortisol levels were also associated with increasing neopterin levels. Interestingly, rather atypical patterns in a diurnal decline of cortisol levels were found during infection periods, such that levels remained raised throughout the day.

**Conclusion:** In conclusion, cortisol increase was related to cellular immune response. Our results suggest that cortisol is a mediator of infectious disease pathogenicity through its impact on the immune system and that wild chimpanzees may be facing energetic stress when sick. By monitoring immune challenges in wild-living animals, our study demonstrates that immune defenses have costs and that these costs are context-specific.

## Introduction

To optimize individual fitness, organisms adopt strategies to improve their ability to survive and reproduce in a fluctuating environment. Due to the necessity of differentially allocating limited resources (e.g., energy), this results in trade-offs between maintenance, growth, and reproduction—leading to species-specific life-history patterns (1).

The activation of the hypothalamic–pituitary–adrenal (HPA) axis in response to stressor results in the systemic elevation of glucocorticoids, which, in mammals, is primarily cortisol (2, 3). An increase of cortisol levels in the bloodstream leads to the rapid mobilization of glucose and contributes toward the restoration of homeostasis by enhancing gluconeogenesis. Therefore, an increase in cortisol provides energy in the face of environmental challenges perceived as stressors (4, 5).

The HPA axis also plays a part in regulating the immune system (2, 3, 6), with stressors associated with immune challenges called immune- or inflammatory stressors. Regulating immune function is essential for the survival of an organism during stressful periods, as well as to modulate immune responses to inflammatory diseases (2). During the acute phase response, immune cells are stimulated through, for example, endotoxins stemming from pathogens, such as bacteria. These immune cells release cytokines that stimulate the HPA axis to release cortisol (7). The increase in blood cortisol levels, in turn, modulates the inflammatory response to pathogens (8). Overall, glucocorticoids have suppressive effects on the maturation, differentiation, and proliferation of immune cells, including innate, T cell, and B cell function (3). Innate immune cells are white blood cells mediating innate immunity, for example, basophils, neutrophils, mast cells, and macrophages. Glucocorticoid receptors are found throughout the immune system and in circulating immune cells, such as macrophages (2, 3, 9). The binding of glucocorticoids to these receptors leads to changes in gene expression and the dysregulation of immune functioning, with severe cortisol-associated immune dysregulation, possibly incurring significant health complications (6).

Immune responses are regulated by antigen-presenting cells such as macrophages/monocytes or dendritic cells, which are components of the innate immunity. Helper T cells are regarded as being the most prolific cytokine producer. They can be subdivided into type 1 helper T cells (Th1) and type 2 helper T cells (Th2). Interferon-gamma is the main Th1 cytokine and produces the pro-inflammatory responses responsible for killing intracellular parasites and for perpetuating autoimmune responses. The Th2 type cytokines include, for example, interleukins 4, 5, 13, and 10, which have more of an anti-inflammatory response (10). As cortisol is involved in inhibiting the interferon-gamma response, an increase in plasma cortisol could induce a decrease in Th1 products, which are produced by type 1 T cells to stimulate macrophages (11). Because activated macrophages/monocytes produce neopterin (12), the measurement of neopterin can be used to monitor cell-mediated immune responses caused, for example, by viral and bacterial infections.

Animals with extended stress responses (chronic stressors) have diminished responses to vaccination and slower wound healing, as well as exacerbated viral and bacterial pathogenesis and altered autoimmune diseases (6). Furthermore, elevated stress levels alter rates of contacts among hosts, pathogens, and vectors on host metabolism and activity levels. For example, an *in vivo* study of viral infection in birds found that, although all individuals became infected after exposure to the virus, only birds with elevated corticosterone levels had viral loads at, or above, the infectious threshold. Moreover, in birds with increased corticosterone levels, the mortality rate was higher than that in controls (13). It was, therefore, hypothesized that elevated glucocorticoid levels put individuals at greater risk from severe illness by affecting their immune system response (6).

The field of ecological immunology (ecoimmunology) aims to understand factors leading to changes in immune system function and, moreover, how these changes affect disease susceptibility in the field and captive settings. A central assumption in ecoimmunology is that mounting an immune response has direct and indirect costs (14). Direct costs include increased metabolic rate and amino acid assimilation, as well as the production of immune proteins involved in the acute phase response. Additional costs of a disease can also occur during infection, such as the development of fever—a key and extremely energy-intensive process and feature of the sickness response (15). The amount of energy required to increase body temperature during the febrile process is considerably high; for example, in humans, to raise the body temperature by 1°C, the metabolic rate needs to be increased by 10–15% (15, 16). Indirect costs of immune system activation include trade-offs with other life-history traits, for example, reduction in growth and reproductive success (14, 17–19). Therefore, to counter against costs incurred during illnesses or periods of short-term energy deficits, an organism may try to conserve energy by downregulating its metabolic rate (5). Sick individuals may also attempt to mitigate costs by increasing their energy resource intake (20) or by adopting sickness behaviors to limit their energy expenditure (21). Behavioral modifications to sickness can be characterized by an overall reduction in physical activity, such as decreased levels of locomotion, sexual behavior, exploration, aggression, food and water intake, and social interest, as well as an increase in sleep duration and shivering to increase heat production (21, 22). Hart (23) proposed that adopting behavioral symptoms of sickness, in combination with the fever response, represents a highly organized strategy to fight infection.

In captive animals, continuous food availability may allow individuals to increase their energy intake, which can cover the costs of immune system activation when sick. It can, therefore, be assumed that energy homeostasis within captive individuals would not be dramatically affected by illness. In contrast, available energy is typically limited in natural environments and, thus, must be allocated among competing physiological processes (14). Moreover, comparing immune system components as an indicator of immune challenges in wild and captive living animals, some blood markers were found to be elevated in wild living birds and dolphins (24, 25). Also, wild chimpanzees experience challenges to their immune system more frequently than captive chimpanzees (26), with severe outbreaks of the respiratory disease reported in the majority of field sites where wild chimpanzees are regularly observed (27–34). Biological similarities and the close genetic relatedness between humans and apes predispose apes to cross-species barrier transmission, and anthroponotic transmission risk is exacerbated due to, for example, tourism (35). Human viruses can cause lethal outbreaks in chimpanzees, even when being nonlethal and mild in humans, indicating a lack of resistance to those viruses in chimpanzees (30, 31, 35). During disease outbreaks, chimpanzees exhibited energy-conserving sickness behaviors, they were found to be less active (29, 35), more lethargic, traveled only short distances, often built day nests, rose late in the morning, and retired early at night (36).

In chimpanzees, physiological changes in, for example, neopterin levels (a marker of cell-mediated immune responses) showed a significant increase concerning sickness behavior, such as extreme fatigue, lethargy, and inappetence (29). However, the extent to which hormones, for example, steroid hormones, may modulate sickness behaviors has only recently been investigated. Furthermore, hormonal correlates of sickness behaviors have yet to be studied in non-human primates within a natural context (e.g., natural infection without treatment, without food provisioning, and exposure to potentially detrimental environmental factors, such as weather and temperature changes) due to the complexities of studying illnesses and immunity outside a controlled clinical setting (37). To study animals under natural conditions is important, given that costs of immunity may be difficult to avoid in a natural environment (14, 18); disease outbreaks in wild living animals can provide an opportunity to test how energy availability and allocation are affected by illness.

Capitalizing on a respiratory disease outbreak in a group of wild habituated chimpanzees in the Taï National Park, Côte d'Ivoire, we investigated, as one of the first studies, how energy availability and allocation are affected by illness by comparing urinary cortisol levels before, during, and after the respiratory disease outbreak. We then tested several predictions related to urinary cortisol changes during the study period as follows. We predicted that cortisol levels increased during the respiratory outbreak to mobilize glucose reserves. Furthermore, we predicted a positive correlation of urinary cortisol and neopterin (a measure of the innate cell-immune response), as mounting an immune response is costly, and sick animals will need to make energy more accessible. Our alternative prediction was that urinary cortisol levels were negatively correlated with urinary neopterin levels, as increasing cortisol levels shift the immune response from the Th1 response to the Th2 response, and neopterin is released from cells that are mainly involved in the Th1 response.

## Materials and Methods

Urine samples were collected from a group of wild chimpanzees before (February 1st to November 12th, 2009), during (November 29th to December 19th, 2009), and after (December 22nd, 2009 to November 16th, 2010) a respiratory outbreak in Taï National Park, Côte d'Ivoire. The respiratory outbreak occurred between November and December 2009 in the south community of chimpanzees who are habituated to human presence and regularly followed since 1994 as part of the Taï Chimpanzee Project (38). Before the outbreak, the south community contained 37 individuals with 18 males (6 adults, 3 adolescents, 3 juveniles, and 6 infants) and 19 females (10 adults, 2 adolescents, and 7 infants) (age–sex class defined from Boesch and Boesch-Achermann) (39). The outbreak lasted from November 26th, when some individuals were observed to exhibit the first signs of respiratory illness (including coughing, sneezing, nasal discharge, and dyspnea) (40), until December 20th, when all surviving individuals ceased to show any signs. During the outbreak, 86% of chimpanzees showed respiratory signs of illness. Diagnostics performed on lung samples from those who died during the outbreak led to the identification of human respiratory syncytial virus A as the main causative pathogen, with coinfections of *Streptococcus pneumoniae* found in some individuals (41). Individuals with particularly severe disease symptoms were treated with a long-acting antibiotic shot (Extencilline, Sanofi-Aventis, France) through remote injection, with 9 of the 12 treated individuals surviving (41). Additional detailed information on the progression of the disease and the pathogen has been published previously (33, 41). Transmission of the human respiratory syncytial virus from humans to apes has often been presumed, but only a few cases have been proven and reported (35, 42, 43). Because there have been several fatal outbreaks of respiratory disease linked to human respiratory viruses in the Taï chimpanzees, extensive health and hygiene measures, quarantine procedures, and behavioral rules, such as wearing face masks, have been applied (44). Moreover, a long-term health monitoring program, as well as a permanent veterinarian on-side, was implemented since the year 2000 (45).

### Sample Analysis

This study included a total of 186 urine samples: 83 samples from 27 individuals (average = 2.6 samples/individual) before the respiratory outbreak, 56 samples from 19 individuals (average = 3.1 samples/individual) during the outbreak, and 47 samples from 20 individuals (average = 2.4 samples/individual) after the outbreak. In eight individuals, urine samples were only collected while symptomatic (e.g., coughing and nasal discharge), whereas five chimpanzees died during the outbreak before a urine sample could be collected, and three chimpanzees remained asymptomatic throughout the outbreak. Urine samples were collected on plastic sheets or leaves and then transferred with a disposable plastic pipette into vials. After collection, urine samples were frozen in liquid nitrogen upon arrival in the camp and finally transported frozen to the Max Planck Institute for Evolutionary Anthropology (MPI-EVA) in Leipzig, Germany. At the MPI-EVA in Leipzig, samples were stored at −80°C before being analyzed.

Urinary cortisol was measured with liquid chromatography–mass spectrometry. For each sample, 10 μl urine was extracted following Hauser (46) with modifications (47). In summary, urine sample extraction included hydrolysis, followed by solid-phase extraction. Afterward, two liquid–liquid extractions were performed, followed by solvolysis and an additional liquid–liquid extraction.

In all samples, urinary neopterin had been previously measured for a study on immune system activation during the same respiratory outbreak (33). To correct for variations in urine dilution, both urinary cortisol and neopterin measures were corrected for specific gravity (SG) using the formula as presented in Miller et al. (48). The SG population average for the wild chimpanzees was 1.017. Urinary cortisol results are expressed in urinary cortisol (nanogram per milliliter) corrected for SG (corr. SG).

Ethical approval was not required for this study because urine samples were collected non-invasively without animal disturbance or harming the chimpanzees.

### Statistical Analyses

To explore factors explaining variation in urinary cortisol levels (nanogram per milliliter corr. SG) in healthy and sick chimpanzees, we ran a linear mixed model (LMM) (49) with a Gaussian error structure and identity link function. The model was fitted in R v.3.4.3 (50) using the R-package lme4, function “lmer” (51). The full model included log-transformed urinary cortisol levels (nanogram per milliliter corr. SG) as the response variable. To test whether urinary neopterin levels (log-transformed) were positively associated with cortisol levels during each of the three periods (before, during, and after the outbreak), we included a sample period (before, during, and after) as interaction with urinary neopterin levels (log-transformed). We further included, as predictor variables, sex and age at the time of sampling (z-transformed) to a mean of zero and a standard deviation of one to achieve comparable estimates (52). To control for externally caused changes in pathogen load, we added the following control variables: sample collection time (z-transformed), survival of the animal (yes or no), and whether the individual had been treated with antibiotics (yes or no). To limit type I error rates to a nominal level of 5% (53, 54), we included individual and sample collection dates as random effects, with random slopes for sample collection date and urinary neopterin levels, respectively. To investigate the significance of each fixed effect, we compared the full model with a null model that excluded the predictor variables while retaining control predictors, as well as the random effects and the random slopes, using a likelihood ratio test (R-function “ANOVA”) (55). A *post hoc* comparison was then performed for each period (before, during, and after) using the function “glht” from the R-package “multcomp” (56, 57).

We assessed the LMM for the required normal distribution and homogeneity of residuals via visual inspection of the q-q plot of the residuals and by plotting residuals against fitted values. The tests revealed no deviation from the assumption. Collinearity was evaluated by determining variance inflation factors (VIF) (58) using the function “vif” from the R-package “car” (59), which revealed that collinearity was not an issue (maximum VIF: 1.2). Model stability was examined by excluding levels of the random effects one at a time from the model and comparing the resulting model estimates for these data with those of the full data set. The results revealed no indication of any influential levels of random effects to exist. Significance for all tests was set to P = 0.05.

As urinary cortisol levels are known to decline throughout the day (60), we included sample collection time as a control variable into the LMM. Surprisingly, in contrast to previous chimpanzee studies (60–63), sample collection time was not a significant effect in our model (*P* = 0.091). To investigate whether this was due to altered diurnal urinary cortisol patterns in sick individuals, we ran a second LMM in which we included the interaction of health status (sick or healthy) with sample collection time as the only test predictor and excluded sample periods from the model, as this would overlap with health status. All other variables, such as urinary neopterin levels, sex, and age at sampling time, survival of the animal (yes or no), and whether the individual had been treated with antibiotics (yes or no), were included as control terms into the second LMM. Random effects were the same as described for the first LMM. We tested all model assumptions, as described earlier. No model assumptions were violated, and collinearity was also not an issue (maximum VIF: 1.5).

## Results

### Cortisol Levels Change During a Respiratory Outbreak Period

**Figure 1:**
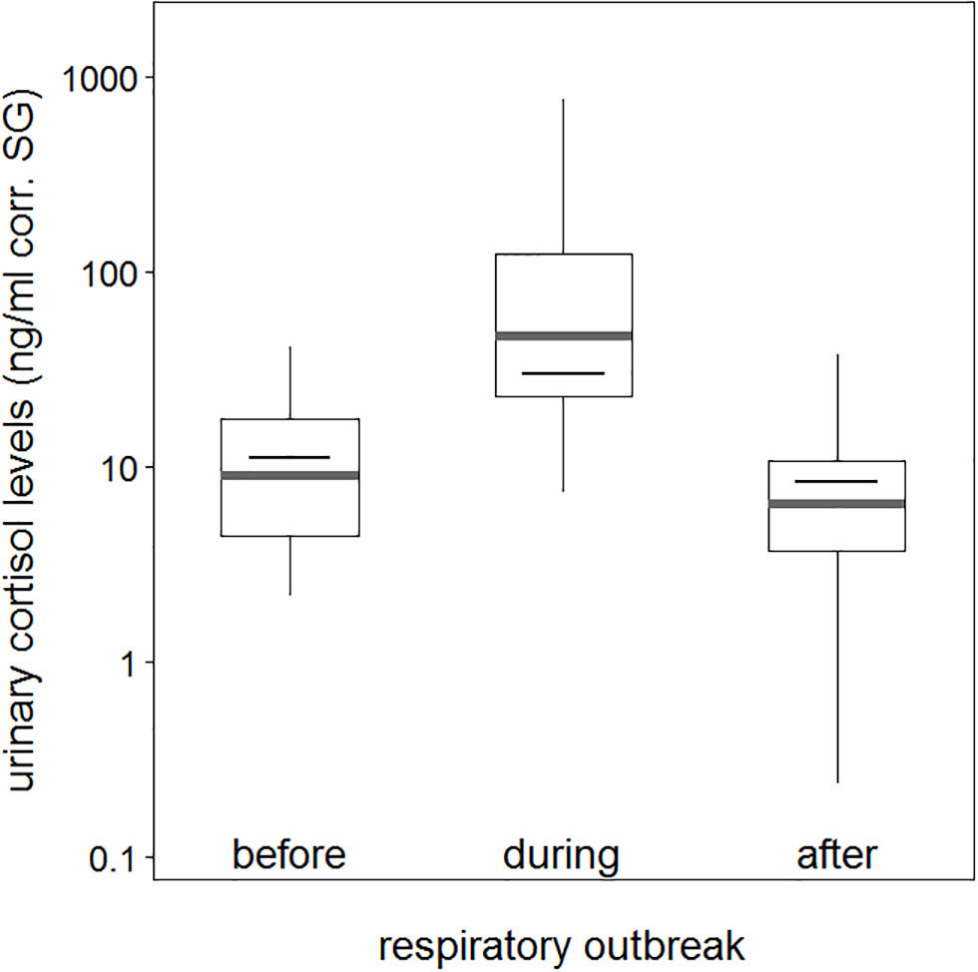
Urinary cortisol levels about sample periods (before, during, and after a respiratory outbreak). Indicated are the median (gray bar) and the fitted model and its 95% confidence intervals (black bar and error bars). Boxes indicate quartiles (25 and 75%), and vertical lines represent quantiles (2.5 and 97.5%). The y-axis is log-transformed.

The average urinary cortisol level was 12.3 (ng/ml corr. SG) before the outbreak, which increased to an average of 140.5 (ng/ml corr. SG) during the outbreak, and declined to an average of 9.7 (ng/ml corr. SG) after chimpanzees did not anymore exhibit respiratory signs of illness (Table 1). Therefore, urinary cortisol levels increased more than 10-fold in chimpanzees with signs of illness in comparison with those in chimpanzees without (Figure 1). Two asymptomatic chimpanzees showed no changes in urinary cortisol levels during the outbreak (Supplementary Figure 1).

**Table 1:** Urinary cortisol levels (ng/ml corr. SG) in wild chimpanzees during each sampling period before, during, and after the respiratory outbreak between November—December 2009.

			**Urinary cortisol (ng/ml corr. SG)**					
**Period**	**Individual** ** (N)**	**Sample** **(N)**	**Average**	**Median**	**Min**.	**Max**.	**Stdev**.	**SE**
Before	27	83	12.3	9.0	0.9	63.3	11.0	1.2
During	19	56	140.5	46.8	6.0	1641.9	264.3	35.3
After	20	47	9.7	6.4	0.1	50.5	10.3	1.5

Comparison of the full model to the null model was significant (χ2 = 114.0, df = 5, *P* < 0.001); however, the interaction between urinary neopterin levels with sample period was not a significant predictor for all three periods (before, during, and after the outbreak), indicating that urinary neopterin and urinary cortisol levels underwent similar changes (Estimate = −0.141, SE = 0.229, *P* = 0.841). Therefore, we ran a reduced model without the interaction term, with both urinary neopterin and sample period as independent predictor variables. The reduced model showed that both urinary neopterin and sample period (before, during, and after) were significant predictors of urinary cortisol levels (Table 2). Urinary cortisol and neopterin levels were positively associated, with increasing neopterin levels corresponding to increasing cortisol levels. No effect was found for sex or age at the time of sampling (Table 2, Supplementary Figure 2). Interestingly, the control variable sample collection time was also not a significant predictor of urinary cortisol levels, although urinary cortisol levels showed a trend in decline as the day progressed, with lower levels found later in the day (Table 2).

**Table 2:** LMM results testing the influence of urinary neopterin, sampling period (before, during, and after the outbreak), age, and sex in sick and healthy wild chimpanzees during a respiratory outbreak on urinary cortisol levels.

**Term**	**Condition**	**Estimates**	**SE**	**CI** **lower**	**CI** **upper**	**χ^2^**	***P***
Intercept		−0.997	0.681	−2.352	0.302		
Sample period:						34.983	** <0.001**
	Before	−1.062	0.209	−1.482	−0.634		
	After	−1.391	0.230	−1.856	−0.942		
Urinary Neopterin		0.692	0.082	0.534	0.859	53.57	** <0.001**
Sex		−0.143	0.215	−0.580	0.276	0.394	0.530
Age at sampling		0.056	0.103	−0.157	0.260	0.279	0.598
Sample collection time		−0.134	0.078	−0.301	0.022	2.862	0.091
Antibiotic treatment		0.203	0.186	−0.151	0.573	1.160	0.281
Survival		−0.093	0.262	−0.583	0.447	0.114	0.736

*Estimates, standard error (SE), as well as lower (CI lower) and upper (CI upper) confidence intervals are included. Significance is indicated in bold*.

A *post hoc* comparison showed that urinary cortisol levels were significantly higher in sick chimpanzees than cortisol levels measured before and after the respiratory outbreak (before vs. during: Estimate = −1.062, SE = 0.209, *P* < 0.001; after vs. during: Estimate = −1.391, SE = 0.230, *P* < 0.001). Urinary cortisol levels before and after the respiratory outbreak were not significantly different (before vs. after: Estimate = 0.329, SE = 0.329, *P* = 0.108).

### Diurnal Variation of Cortisol Levels

**Figure 2:**
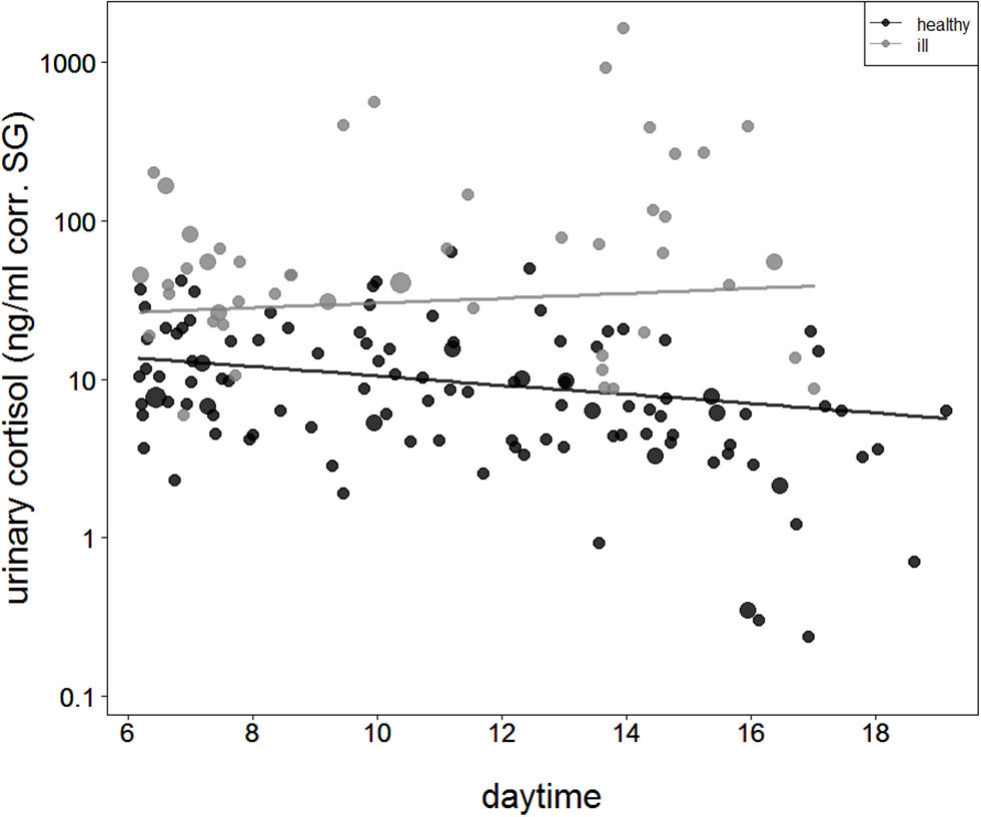
Urinary cortisol levels concerning sample collection time during asymptomatic periods (healthy = filled black circles and black straight line) and during the respiratory outbreak (ill = filled gray circles and straight gray line). The y-axis is log-transformed.

The comparison of the second full model to the null model was also significant (full-null model comparison: χ2 = 40.9, df = 3, *P* < 0.001), with the interaction between health status and sample collection time as the only effect tested. During asymptomatic periods before and after the outbreak, urinary cortisol levels were found to decline with sample collection time. However, when chimpanzees showed signs of respiratory disease, this pattern was no longer visible (Figure 2).

## Discussion

In wild habituated chimpanzees, urinary cortisol levels increased when individuals exhibited signs of respiratory disease. These increases in cortisol levels were found to be related to the innate immune response as urinary neopterin levels increased positively with cortisol levels. Finally, although urinary cortisol levels showed the expected diurnal decline when chimpanzees were asymptomatic, interestingly, the diurnal decline was no longer significant when chimpanzees experienced respiratory signs of illness.

In our study, urinary cortisol showed a 10-fold increase during illness. Concerning other changes in urinary cortisol levels in chimpanzees, this is five times more than the nearly 2-fold increase, comparing periods of unstable vs. with stable male dominance relationship periods (62) or control days with hunting days or days with intergroup encounters (64). The comparably substantial increase in cortisol levels found in this study indicates that sick chimpanzees drastically increased glucose availability in their blood.

During the outbreak, increased cortisol levels in the wild chimpanzees may also indicate that they may have experienced fever, as it has been previously shown in goats, guinea pigs, and humans that fever always accompanied an increase in cortisol levels (7, 65, 66). Additionally, in humans, fever is energetically costly, as raising the body temperature requires increasing the basal metabolic rate (67). As the immune response requires energy for optimal functioning (68), it is possible that the wild chimpanzees were not able to compensate energy expenditure by increasing their energy intake while ill. As a consequence, cortisol secretion increased during this period, as glucocorticoids are essential for mobilizing stored energy resources (69). Additionally, certain sickness behaviors may further decrease the available energy budget, such as lower rates of foraging and, therefore, energy intake. Behavioral strategies may exist to conserve energy expenditure, for example, it has been previously shown in primates that when parasite infections were high, resting rates increase, whereas more costly behaviors (e.g., grooming and copulation) decrease (70, 71). In chimpanzees, energy may be restricted during periods of illness. For example, in Kanyawara chimpanzees during a respiratory outbreak, urinary c-peptide levels, a marker of energy balance, were found to decline despite favorable feeding conditions (72). Furthermore, during respiratory outbreaks in both Taï and Gombe chimpanzee populations, daily food intake decreased, as well as overall activity and travel distances (35, 36, 40, 73). It is, therefore, hypothesized that these sickness behaviors might help to conserve energy during an illness.

Wild animals may mount a greater cortisol response than captive animals. At the same time, wild animals are not typically provisioned with medications and food and water and still need to travel, climb, and forage for food, even when ill. In captive animals, however, an increase in energy intake through regular provisioning may allow them to cover the costs of mounting an immune response when ill. In captivity, sick animals also have shorter, or no, travel distances to forage for food, as it is usually provided in closer proximity. Therefore, during an illness, it can be assumed that energy homeostasis within a captive individual would be less affected than within wild animals during an illness. Cortisol levels were found to be stable in modern western humans during an experimental immune challenge. During this immune challenge, subjects were found to increase their caloric intake, which may have offset the energy costs incurred from mounting an immune response (37).

However, energetic stress may not be the exclusive explanation for elevated urinary cortisol levels during illness. Another factor could also be social stress as a consequence of isolation, as chimpanzees are highly social animals. During illness, a chimpanzee becomes isolated, often traveling at a slower pace than the group or spending extended periods of nesting (39, 71, 74). May those behavioral changes also help to conserve energy; however, self-distancing may also increase their psychological stress level; for example, this reduces the possibility of social buffering (75) and also increases predation risk (76).

Changes in the diurnal cortisol pattern support the prediction that wild chimpanzees mobilize glucose reserves when ill and, therefore, have increased urinary cortisol levels. As observed in healthy chimpanzees during our study, urinary cortisol levels were found to decline during the day—a characteristic pattern found in healthy chimpanzees (60–63), gorillas (77, 78), macaques (79), and humans (77, 80). The diurnal decline in urinary cortisol levels during days without symptoms is similar to the general pattern of diurnal decline observed in chimpanzees at this site (63). However, in chimpanzees displaying signs of a respiratory infection, urinary cortisol levels remained elevated throughout the day. A loss or disturbance of diurnal decline in cortisol levels has also been reported in humans with various disorders, including chronic fatigue syndrome, anxiety disorders, fibromyalgia, rheumatoid arthritis, depression, bipolar disorders, and respiratory diseases (81, 82). Moreover, the slope of diurnal cortisol levels throughout the day was also found to be flatter (83).

By monitoring immune challenges in wild-living animals, our study demonstrates that immune defenses have costs. Given the costs of living in a natural environment with fluctuating resources and in the face of immune challenges, understanding energy allocation between growth, maintenance, and health, as well as the interplay between immune function, sickness behaviors, and endocrinological correlates, is garnering increasing research interest. Here, we show that cortisol may play a role in mediating the innate immune response, with sick individuals exhibiting higher levels of both urinary cortisol and neopterin during an outbreak. Additionally, sick individuals did not display the typical healthy pattern of diurnal decline in cortisol levels—but rather, raised levels throughout the day. Although energetic stress seems to play a role in increasing urinary cortisol during periods of illness, other factors, such as psychological or social stress, should not be discounted. Further examination of sickness behaviors with other endocrinological, immunological, or energetic biomarkers is required to understand better the adoption of various life-history patterns and how organisms may modulate their physiological processes to increase their survival and fitness.

## Data Availability Statement

The raw data supporting the conclusions of this article will be made available by the authors, without undue reservation.

## Ethics Statement

Ethical approval was not required for this study in line with institutional guidelines and local legislation, because urine samples were collected non-invasively without animals' disturbance or harming the chimpanzees.

## Author Contributions

VB, DW, RW, and TD: conception and design. CC, RW, and TD: sample acquisition. VB and AP: statistical analysis. All authors were involved in the interpretation of the data. VB and TD: drafting of the manuscript. All authors revised, reviewed and approved the final version of the manuscript.

## Conflict of Interest

The authors declare that the research was conducted in the absence of any commercial or financial relationships that could be construed as a potential conflict of interest.
