# Supplementary material for: Urinary Cortisol Increases During a Respiratory Outbreak in Wild Chimpanzees
Source: Front Vet Sci. 2020 Aug 21;7:485. doi: 10.3389/fvets.2020.00485 (PMC7472655; doi:10.3389/fvets.2020.00485)
Supplement: Supplementary file 1 [file Image_1.pdf]

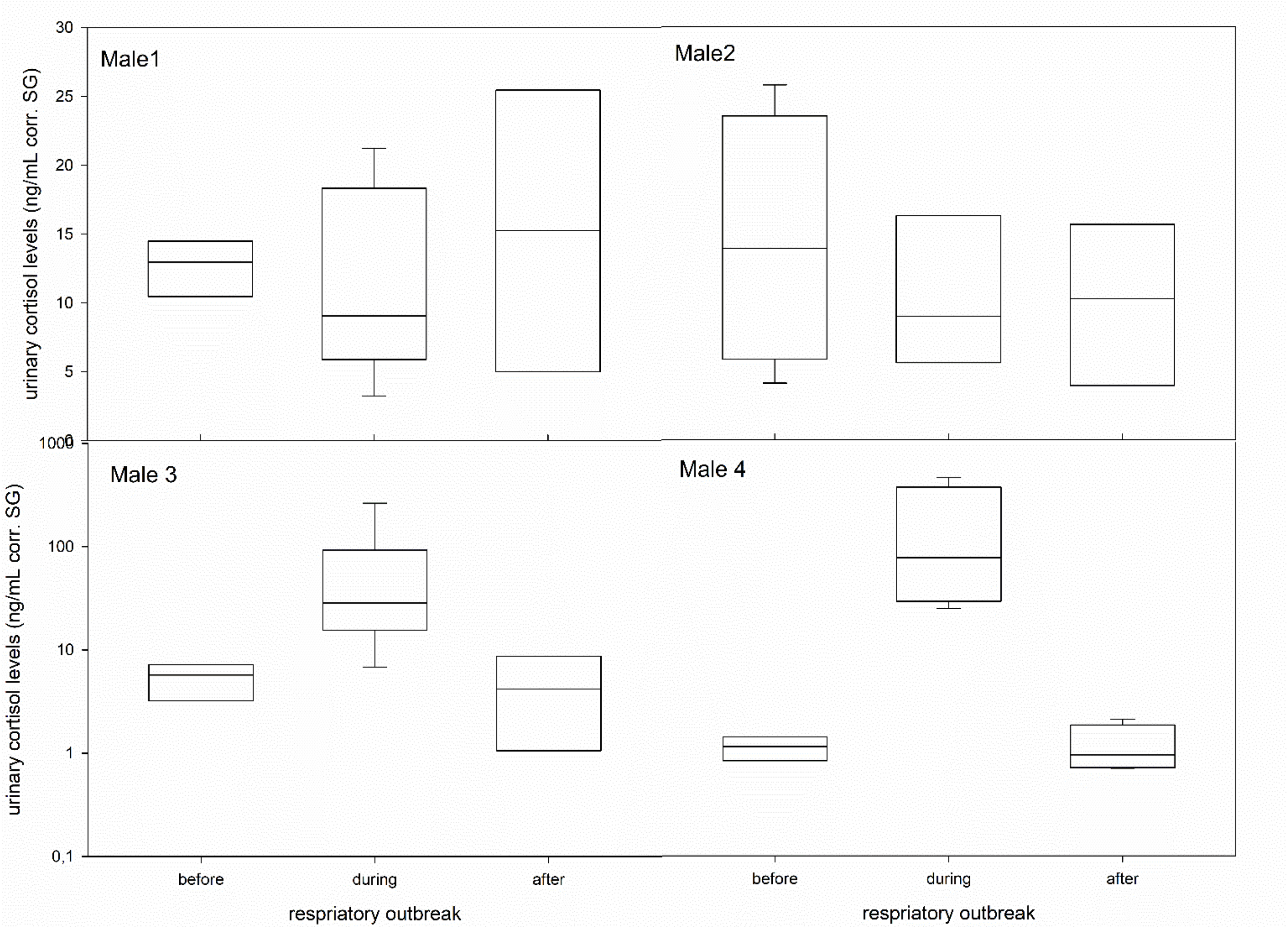

Supplement Figure 1: Urinary cortisol levels of two asymptomatic chimpanzee males (males 1 and 2) and two males with sickness symptoms (males 3 and 4) in relation to sample periods (before, during and after a respiratory outbreak). Indicated are the median (black bar). Boxes indicate quartiles (25 and 75%) and vertical lines represent quantiles (2.5 and 97.5%). Y-axis for the two males with symptoms were log transformed.
