# Supplementary material for: Urinary Cortisol Increases During a Respiratory Outbreak in Wild Chimpanzees
Source: Front Vet Sci. 2020 Aug 21;7:485. doi: 10.3389/fvets.2020.00485 (PMC7472655; doi:10.3389/fvets.2020.00485)
Supplement: Supplementary file 2 [file Image_2.pdf]

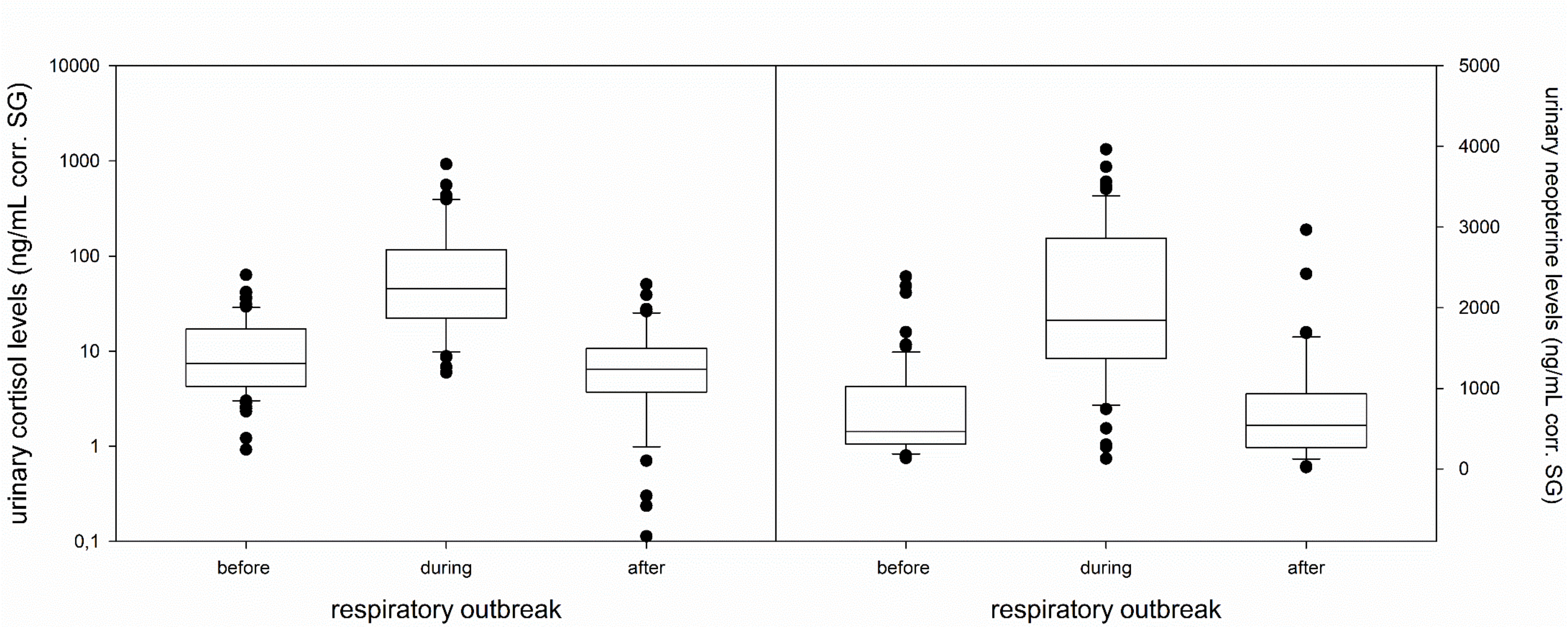

Supplement Figure 2: Urinary cortisol levels (left hand side) and urinary neopterin levels (right hand site) in relation to sample periods (before, during and after a respiratory outbreak). Indicated are the median (black bar). Boxes indicate quartiles (25 and 75%) and vertical lines represent quantiles (2.5 and 97.5%). The cortisol y-axis is log-transformed.
